# Supplementary material for: IntelliProfiler: a research workflow for analyzing multiple animals with a high-resolution home-cage RFID system
Source: Lab Anim (NY). 2026 Jan 16;55(2):48–63. doi: 10.1038/s41684-025-01668-4 (PMC12867760; doi:10.1038/s41684-025-01668-4)
Supplement: Supplementary file 2 — Reporting Summary [file 41684_2025_1668_MOESM2_ESM.pdf]

nature portfolio

Corresponding author(s): Noriko Osumi, Shohei Ochi

Last updated by author(s): Nov 30, 2025

Reporting Summary

Nature Portfolio wishes to improve the reproducibility of the work that we publish. This form provides structure for consistency and transparency in reporting. For further information on Nature Portfolio policies, see our Editorial Policies and the Editorial Policy Checklist.

Statistics

For all statistical analyses, confirm that the following items are present in the figure legend, table legend, main text, or Methods section.

n/a	Confirmed	
☐	☒	The exact sample size (n) for each experimental group/condition, given as a discrete number and unit of measurement
☐	☒	A statement on whether measurements were taken from distinct samples or whether the same sample was measured repeatedly
☐	☒	The statistical test(s) used AND whether they are one- or two-sided<br>*Only common tests should be described solely by name; describe more complex techniques in the Methods section.*
☒	☐	A description of all covariates tested
☐	☒	A description of any assumptions or corrections, such as tests of normality and adjustment for multiple comparisons
☐	☒	A full description of the statistical parameters including central tendency (e.g. means) or other basic estimates (e.g. regression coefficient) AND variation (e.g. standard deviation) or associated estimates of uncertainty (e.g. confidence intervals)
☐	☒	For null hypothesis testing, the test statistic (e.g. F, t, r) with confidence intervals, effect sizes, degrees of freedom and P value noted<br>*Give P values as exact values whenever suitable.*
☒	☐	For Bayesian analysis, information on the choice of priors and Markov chain Monte Carlo settings
☒	☐	For hierarchical and complex designs, identification of the appropriate level for tests and full reporting of outcomes
☐	☒	Estimates of effect sizes (e.g. Cohen's d, Pearson's r), indicating how they were calculated

Our web collection on statistics for biologists contains articles on many of the points above.

Software and code

Policy information about availability of computer code

Data collection	Behavioral data were acquired using a commercially available high-resolution radio frequency identification (RFID) floor plate (Phenovance, Japan) composed of 96 antenna tiles (5 × 5 cm) arranged in a 12 x 8 grid. RFID tags (Phenovance, Japan) were subcutaneously implanted in the abdominal region of individual mice under anesthesia. Raw logs (timestamps, antenna ID, and transponder ID) were streamed via USB and captured with TeraTerm software. Data were binned to 1-s intervals and converted to X-Y coordinates using the IntelliProfiler workflow. When multiple detections occurred within the same 1-s bin, the first valid X-Y coordinate was retained (deterministic rule). Missing coordinates were imputed by the last observation carried forward (LOCF), as described in the "Methods" section and implemented in the R script (IP_general.R).
Data analysis	Data processing and analyses were conducted in R (version 4.2.2) and Python (version 3.9.7). Antenna IDs were mapped to X-Y coordinates, and Euclidean distances were used to compute per-mouse travel distances. Social proximity was quantified using the Close Contact Ratio (CCR; proportion of time spent in close proximity to conspecifics, as defined in Methods). Unsupervised structure was explored with principal component analysis (PCA) and clustering; the number of clusters was guided by elbow and silhouette criteria. Network representations were generated in Cytoscape (version 3.9.1), using CCR values serving as edge weights. Custom scripts for preprocessing, statistics, and visualization are available in the IntelliProfiler workflow in GitHub repository: https://github.com/ShoheiOchi/IntelliProfiler.

For manuscripts utilizing custom algorithms or software that are central to the research but not yet described in published literature, software must be made available to editors and reviewers. We strongly encourage code deposition in a community repository (e.g. GitHub). See the Nature Portfolio guidelines for submitting code & software for further information.

Data

Policy information about availability of data

All manuscripts must include a data availability statement. This statement should provide the following information, where applicable:

- Accession codes, unique identifiers, or web links for publicly available datasets
- A description of any restrictions on data availability
- For clinical datasets or third party data, please ensure that the statement adheres to our policy

The behavioral datasets generated and analyzed during the current study are available from the corresponding author upon reasonable request. The analysis code and input data used to generate the figures are available at the IntellliProfiler GitHub repository: https://github.com/ShoheiOchi/IntelliProfiler.

Research involving human participants, their data, or biological material

Policy information about studies with human participants or human data. See also policy information about sex, gender (identity/presentation), and sexual orientation and race, ethnicity and racism.

Reporting on sex and gender	*Use the terms sex (biological attribute) and gender (shaped by social and cultural circumstances) carefully in order to avoid confusing both terms. Indicate if findings apply to only one sex or gender; describe whether sex and gender were considered in study design; whether sex and/or gender was determined based on self-reporting or assigned and methods used. Provide in the source data disaggregated sex and gender data, where this information has been collected, and if consent has been obtained for sharing of individual-level data; provide overall numbers in this Reporting Summary. Please state if this information has not been collected. Report sex- and gender-based analyses where performed, justify reasons for lack of sex- and gender-based analysis.*
Reporting on race, ethnicity, or other socially relevant groupings	*Please specify the socially constructed or socially relevant categorization variable(s) used in your manuscript and explain why they were used. Please note that such variables should not be used as proxies for other socially constructed/relevant variables (for example, race or ethnicity should not be used as a proxy for socioeconomic status). Provide clear definitions of the relevant terms used, how they were provided (by the participants/respondents, the researchers, or third parties), and the method(s) used to classify people into the different categories (e.g. self-report, census or administrative data, social media data, etc.) Please provide details about how you controlled for confounding variables in your analyses.*
Population characteristics	*Describe the covariate-relevant population characteristics of the human research participants (e.g. age, genotypic information, past and current diagnosis and treatment categories). If you filled out the behavioural & social sciences study design questions and have nothing to add here, write "See above."*
Recruitment	*Describe how participants were recruited. Outline any potential self-selection bias or other biases that may be present and how these are likely to impact results.*
Ethics oversight	*Identify the organization(s) that approved the study protocol.*

Note that full information on the approval of the study protocol must also be provided in the manuscript.

Field-specific reporting

Please select the one below that is the best fit for your research. If you are not sure, read the appropriate sections before making your selection.

☒ Life sciences ☐ Behavioural & social sciences ☐ Ecological, evolutionary & environmental sciences

For a reference copy of the document with all sections, see nature.com/documents/nr-reporting-summary-flat.pdf

Life sciences study design

All studies must disclose on these points even when the disclosure is negative.

Sample size	No priori sample-size calculation was performed. The minimal group size was set at four to exceed the common statistical minimum ($n \geq 3$) with an additional buffer for potential exclusion. The maximum group size was capped at 16 to match the physical capacity of the home-cage setup. A group size of eight was adopted in several experiments as pragmatic midpoint (Twice the minimum and half the maximum).
Data exclusions	During data collection in the 16-male cohort, one RFID tag was dislodged; data from that mouse were excluded. Aged female mice were not included because they could not be sourced from the same vendor (CLEA Japan, Inc.) during the study period.
Replication	Independent experimental replication was not performed.
Randomization	C57BL/6J wild-type mice (CLEA Japan, Inc.) were purchased without pre-selection and ramdomly assigned to experimental groups (8-week old groups of four, eight, and 15-16 male and female mice; 53-week old groups of four and eight males). For the ASD model analysis, 8-week-old aged father-derived offspring (AFO) mice with similar body weights were selected from the same cohort (multiple dams mated with aged fathers) to minimize within-group variability.

Blinding	After randomized placement into the home cages, animals were not handled by investigators for the entire recording period. Behavioral data acquisition and primary preprocessing were fully automated (high-resolution RFID floor plate + scripted pipeline). Investigators and primary analysts did not access group labels during data collection or initial processing; analyses were performed on anonymized animal IDs, and group allocation codes were revealed only after per-animal metrics had been computed to run statistical comparisons and prepare figures. No manual scoring was performed. Accordingly, data collection proceeded under an effectively blinded workflow that minimizes observer bias.

Reporting for specific materials, systems and methods

We require information from authors about some types of materials, experimental systems and methods used in many studies. Here, indicate whether each material, system or method listed is relevant to your study. If you are not sure if a list item applies to your research, read the appropriate section before selecting a response.

Materials & experimental systems

n/a	Involved in the study
☒	☐ Antibodies
☒	☐ Eukaryotic cell lines
☒	☐ Palaeontology and archaeology
☐	☒ Animals and other organisms
☒	☐ Clinical data
☒	☐ Dual use research of concern
☒	☐ Plants

Methods

n/a	Involved in the study
☒	☐ ChIP-seq
☒	☐ Flow cytometry
☒	☐ MRI-based neuroimaging

Animals and other research organisms

Policy information about studies involving animals; ARRIVE guidelines recommended for reporting animal research, and Sex and Gender in Research

Laboratory animals	Male and female C57BL/6J wild-type (WT) mice at 7-week-old and male WT mice at 52 week-of-old were obtained from CLEA Japan, Inc. AFO mice were generated at Tohoku University by mating these male mice with 8–12-week-old females. For AFO controls, 13–32-week-old males were mated with 10-12-week-old females at CLEA Japan; their offspring were used as controls. Male and female AFO mice were weaned at 4-week old and housed in littermate groups (2-5 animals per cage) until experiments.
Wild animals	Not applicable.
Reporting on sex	Both males and females were included for wild-type and AFO cohorts. For aged cohorts, only males were included due to vendor unavailability of aged females from the same source.
Field-collected samples	Not applicable.
Ethics oversight	All experimental procedures were approved by the Ethics Committee for Animal Experiments at the Tohoku University Graduate School of Medicine (approval no. 2021MdA-020-13).

Note that full information on the approval of the study protocol must also be provided in the manuscript.

Plants

Seed stocks	*Report on the source of all seed stocks or other plant material used. If applicable, state the seed stock centre and catalogue number. If plant specimens were collected from the field, describe the collection location, date and sampling procedures.*
Novel plant genotypes	*Describe the methods by which all novel plant genotypes were produced. This includes those generated by transgenic approaches, gene editing, chemical/radiation-based mutagenesis and hybridization. For transgenic lines, describe the transformation method, the number of independent lines analyzed and the generation upon which experiments were performed. For gene-edited lines, describe the editor used, the endogenous sequence targeted for editing, the targeting guide RNA sequence (if applicable) and how the editor was applied.*
Authentication	*Describe any authentication procedures for each seed stock used or novel genotype generated. Describe any experiments used to assess the effect of a mutation and, where applicable, how potential secondary effects (e.g. second site T-DNA insertions, mosiacism, off-target gene editing) were examined.*
